# Urethane Formation with an Excess of Isocyanate or Alcohol: Experimental and Ab Initio Study

**DOI:** 10.3390/polym11101543

**Published:** 2019-09-22

**Authors:** Wafaa Cheikh, Zsófia Borbála Rózsa, Christian Orlando Camacho López, Péter Mizsey, Béla Viskolcz, Milán Szőri, Zsolt Fejes

**Affiliations:** Institute of Chemistry, University of Miskolc, Miskolc-Egyetemváros A/2, H-3515 Miskolc, Hungary; cheikhwafaa.92@gmail.com (W.C.); kemzsofi@uni-miskolc.hu (Z.B.R.); chrisscamacho@gmail.com (C.O.C.L.); kemizsey@uni-miskolc.hu (P.M.); bela.viskolcz@uni-miskolc.hu (B.V.)

**Keywords:** urethane formation, isocyanate excess, mechanism, ab initio, allophanate, kinetics

## Abstract

A kinetic and mechanistic investigation of the alcoholysis of phenyl isocyanate using 1-propanol as the alcohol was undertaken. A molecular mechanism of urethane formation in both alcohol and isocyanate excess is explored using a combination of an accurate fourth generation Gaussian thermochemistry (G4MP2) with the Solvent Model Density (SMD) implicit solvent model. These mechanisms were analyzed from an energetic point of view. According to the newly proposed two-step mechanism for isocyanate excess, allophanate is an intermediate towards urethane formation via six-centered transition state (TS) with a reaction barrier of 62.6 kJ/mol in the THF model. In the next step, synchronous 1,3-H shift between the nitrogens of allophanate and the cleavage of the C–N bond resulted in the release of the isocyanate and the formation of a urethane bond via a low-lying TS with 49.0 kJ/mol energy relative to the reactants. Arrhenius activation energies of the stoichiometric, alcohol excess and the isocyanate excess reactions were experimentally determined by means of HPLC technique. The activation energies for both the alcohol (measured in our recent work) and the isocyanate excess reactions were lower compared to that of the stoichiometric ratio, in agreement with the theoretical calculations.

## 1. Introduction

Isocyanates are among the most valued synthetic intermediates [1]. Their reactions with various nucleophiles give rise to important classes of compounds, such as urethanes, thiouretanes and ureas. These reactions are of industrial importance because they provide the basis of the very versatile class of polymers, polyurethanes, where the main process is the reaction of di-isocyanates with polyols.

From a kinetic and mechanistic point of view, the addition reaction between the isocyanato and the hydroxyl group has been of interest since the 1930 [2]. The first detailed kinetic investigations of uncatalyzed and catalyzed reactions was made by Baker and co-workers in the 1940s [3,4,5]. Their studies concluded that the apparently bimolecular addition is catalyzed by both the alcohol reactant and the urethane product [4]. Later, the possibility of having alcohol associates as the active reacting partner was pointed out [6,7,8]. The rate constant of the reaction strongly depends on the solvent [9].

**Figure 1:**
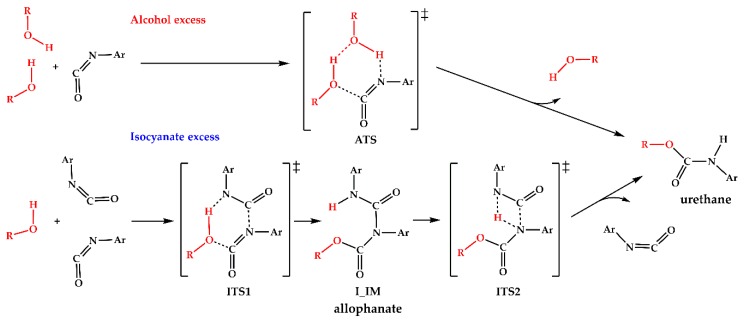
Elementary reaction mechanism for urethane bond formation. The alcohol excess mechanism (top) involves a hydrogen-bonded alcohol associate as the reactant, while the isocyanate excess mechanism (bottom) starts with dipole-dipole stabilized intermolecular isocyanate dimer. In the present study R = Pr and Ar = Ph.

The experimental activation energies for the reactions of aryl isocyanates with alcohols are generally in the range of 17–54 kJ/mol ([10,11] and references cited in each). For a given reaction the activation energy depends on the solvent and the ratio of the reactants. Theoretical calculations showed that the rather high energy barrier (>100 kJ/mol) [8,12,13] needed for reaching the bimolecular transition state (direct addition) becomes substantially lower if one or two additional alcohol molecules (alcohol catalysis), or a urethane molecule (autocatalysis) are also incorporated into the transition state [8,14,15]. A schematic mechanism of such alcohol catalysis is presented in the upper part of Figure 1.

Strong intermolecular hydrogen bonds can stabilize these alcohol associates which is also confirmed by consistent molecular dynamic simulation and X-ray experiment study on liquid 1-propanol by Akiyama and co-workers [16]. This fact makes the above-mentioned mechanism plausible in the condition of excess alcohol. On the other hand, isocyanates also have potential to form associates due to its large permanent electric dipole moment (|μ_tot,MP2/aug-cc-pVTZ_| = 2.78 D [17], |μ_tot,MW_| = 2.81 D [18]). Indeed, Lenzi et al. reported interaction energy of 24.3–32.8 kJ/mol for alkyl-isocyanates dimers using density functional theory (DFT) calculation [19]; therefore, these isocyanate associates can also be formed in isocyanate excess and can provide a starting point for urethane formation. The proposed reaction mechanism can be seen in the bottom of Figure 1. In this paper, we present this new possible reaction mechanism, supported by both theoretical and experimental findings, in which two isocyanate molecules facilitate the urethane formation process. The theoretical investigation of the reaction mechanism requires an adequate and robust quantum chemical protocol. The fourth generation G4MP2 quantum chemical protocol had been demonstrated several times [20,21,22] to provide overall thermodynamic results with chemical accuracy [23].

## 2. Materials and Methods

### 2.1. Materials

The reaction of phenyl isocyanate (PhNCO) and 1-propanol (PrOH) was conducted at a stoichiometric ratio and at 20-fold isocyanate molar excess. PhNCO (≥99%, Acros Organics BVBA, Geel, Belgium) was used as received. Acetonitrile (ACN) was HPLC grade (VWR International LLC, Debrecen, Hungary). To achieve low water content, PrOH (≥99%, VWR International LLC, Debrecen, Hungary) and tetrahydrofuran (THF) (≥99%, VWR International LLC, Debrecen, Hungary) were stored over 20%(m/V) activated molecular sieves (3Å, beads, VWR International LLC, Debrecen, Hungary) for at least two days [24]. *n*-Butylamine (≥99%) was purchased from Merck Kft. (Budapest, Hungary). *N*,*N′*-diphenylurea (≥98%) was purchased from Alfa-Aesar (Ward Hill, MA, USA).

### 2.2. Kinetic Experiments

Stock solutions of 2.0 M PhNCO and 2.0 M PrOH in THF (for the stoichiometric runs), and 4.0 M PhNCO and 0.2 M PrOH in THF (for the NCO excess runs) were prepared in volumetric flasks. From the prethermostated (±0.1 °C) stock solutions, 5.0 mL of PhNCO and 5.0 mL of PrOH solutions were pipetted into a prethermostated glass vial, which was then capped. The experiments were conducted at 303,313 and 323 K. At different time intervals a sample of 10 µL was withdrawn from the reaction mixture and mixed into 990 µL ACN containing 30 µL of *n*-butylamine in order to quench the reaction. The amine reacted spontaneously with the isocyanate to form the adduct *N*-butylphenylurea. The quenched samples were further diluted by a factor of 50 (for the PhNCO excess runs) or 5 (for the stoichiometric runs) with an ACN:H_2_O = 1:1 mixture and were subjected to HPLC analysis. The concentration of the *N*,*N’*-diphenylurea side-product (originating from the hydrolysis of PhNCO) was also determined and was found to be a maximum of 5.6% of the starting PhNCO concentration.

### 2.3. Analysis Method

Analysis of the quenched and diluted samples was done using a Shimadzu HPLC (Shimadzu Corporation, Kyoto, Japan) equipped with LC-20AD pumps, SIL-20AC autosampler, DGU-20A3R degassing unit, CTO-20A column oven and a SPD-M20A photodiode array detector. A SunShell C8 column (2.6 µm, 150 × 3.0 mm; ChromaNik Technologies Inc., Osaka, Japan) thermostated at 40 °C was used for the separation. The injection volume was 25 µL. The eluent was ACN:H_2_O with a gradient as follows: 0–3.50 min, 42% ACN; 3.51–4.50 min, 82% ACN; 4.51–9.00 min and 42% ACN, at a flow rate of 0.6 mL/min. The product *n*-propyl phenylcarbamate was quantified at 239 nm. For calibration, the reference compound was synthesized from PhNCO in PrOH and purified by flash chromatography.

### 2.4. Theoretical Method

G4MP2 composite method [23] was applied for obtaining accurate thermodynamic properties, such as zero-point corrected relative energy (Δ*E*_0_), relative enthalpy (Δ*H*(T)) and relative molar Gibbs free energy (Δ*G*(T,P)) for the species involved in the studied reaction mechanisms. As part of G4MP2 protocol B3LYP [25], functional was applied in combination with the 6–31G(2df,p) (this basis set is noted as GTBas3 in Gaussian09 [26]) basis set for Berny algorithm driven geometry optimizations (using “tight” convergence criteria with the following thresholds: maximum force = 0.000015, RMS force = 0.000010, maximum displacement = 0.000060 and RMS displacement = 0.000040) and frequency calculations. Normal mode analysis was performed on the optimized structures at the same level of theory to characterize their identities on the potential energy surface (PES). TS structures were also checked by visual inspection of the intramolecular motions corresponding to the imaginary wavenumber using GaussView05 [27] and were confirmed by intrinsic reaction coordinate (IRC) calculations [28] for mapping out the minimal energy pathways (MEP).

For each step of the G4MP2 protocol, including geometry optimization and single point calculations, the SMD polarizable continuum model [29] was used to mimic the effect of the surrounding solvent of 1-propanol (PrOH, *ε*_r_ = 20.524) as well as that of tetrahydrofuran (THF, *ε_r_* = 7.4257). It is worthy to note that the static relative permittivity for phenyl isocyanate (PhNCO, *ε*_r_ = 8.940 [30]) is close to that of THF; therefore, the potential energy surface (PES) obtained in PhNCO and in THF can be expected to be similar. The SMD model is considered highly accurate, since it achieves mean unsigned errors of 2.5–4.1 kJ/mol in the solvation free energies of neutral species [29] for the reported test set. All quantum chemical calculations were performed by the Gaussian09 [26] software package. The optimized structures and calculated G4MP2 thermochemical properties (*E*_0_, *H*(298.15 K) and *G*(298.15 K, 1 atm)) are collected in the Tables S1 and S2 of the Supporting Information.

## 3. Results and Discussion

### 3.1. Results of the Kinetic Experiments

The rate constants (*k_S_* for the stoichiometric reaction, *k_I,obs_* for the reaction running at 20-fold isocyanate excess) at different temperatures were determined by plotting the urethane concentration against time (Figure 1) and applying a non-linear regression using the kinetic Equation (1) for second order and Equation (2) for pseudo first-order reactions. For the latter, because of the 20-fold isocyanate excess, the isocyanate concentration during the reaction was regarded to be constant ([PhNCO]_0_). In like manner, the rate constant *k_I_* can be calculated from the observed rate constant *k_obs_* (Equation (3)).

(1) $$\left[urethane\right]={\left[PrOH\right]}_{0}\times \left(1-\frac{1}{1+{\left[PrOH\right]}_{0}\times k_{S}\times t}\right)$$

(2) $$\left[urethane\right]={\left[PrOH\right]}_{0}\times \left(1-e^{-k_{I,obs}\times t}\right)$$

(3) $$k_{I,obs}=k_{I}\times {\left[PhNCO\right]}_{0}$$

**Figure 2:**
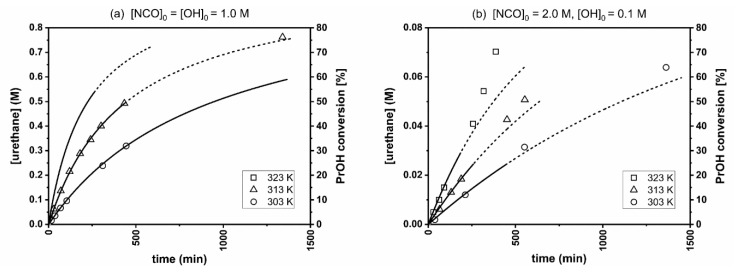
Experimental kinetic curves. (**a**) Second-order kinetics for the stoichiometric ratio. (**b**) pseudo first-order kinetics for the 20-fold PhNCO excess. Data points used for fitting and reaction rate constants’ determinations are indicated by solid curve segments.

It is apparent from Figure 2 that the first few data points fit well for the appropriate equations, namely, the second order one (Equation (1)) for Figure 2a and the pseudo first-order one (Equation (2)) for Figure 2b, but at later reaction stages a positive deviation occurs which possibly accounts from urethane autocatalysis. In case of a stoichiometric NCO/OH ratio, the addition can be described with second-order kinetics up to 50–60% conversion. When the isocyanate is in 20-fold excess, the reaction follows pseudo first-order kinetics only up to a conversion of 25–30%. Therefore, only the initial domain of the data (see Figure 2) were used for non-linear regressions and reaction rate constant calculations.

Table 1 summarizes the kinetic parameters of the reactions. For the alcohol excess reaction, the rate constants (*k_A_*) and the activation energies were measured in our previous work [11]. Both at alcohol excess and at isocyanate excess the Arrhenius activation energies are lower than that of the stoichiometric reaction. (For the Arrhenius plots see Figure S1 in Supporting Information.) From this it is assumed that not only alcohol, but isocyanate molecules can also exert a catalytic effect and facilitate the reaction. At or near stoichiometric ratios, both self-catalytic pathways can occur.

Rate constants in Table 1 are apparent rate constants, as the values depends on reaction conditions, such as the applied solvent and the concentrations of the reactants.

### 3.2. Results of the Theoretical Calculations

**Figure 3:**
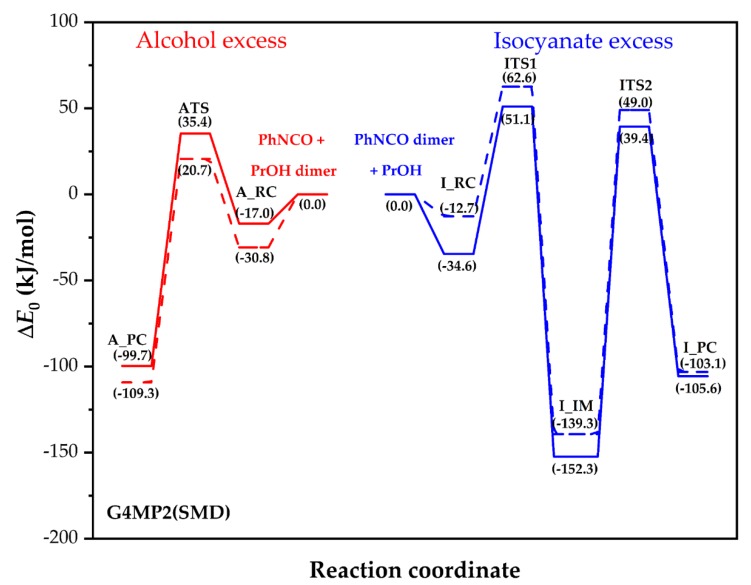
G4MP2 energy profiles (zero-point corrected) for the alcoholic route in solvents 1-PrOH (red solid line) and THF (red dashed line), and for the isocyanate route with 1-PrOH (blue solid line) and THF (blue dashed line).

Hydrogen bond stabilized alcohol associates have been confirmed [16] and their role of reduction of the activation barrier in the urethane formation is already accepted [8]. Therefore, the energies of the PrOH dimer and PhNCO were used as the references in this G4MP2 model calculation. Thermodynamic values for the stationary points of the reactive potential energy surface are summarized in Table 2 and relative zero-point corrected energies in PrOH and THF are also displayed in Figure 3.

**Figure 4:**
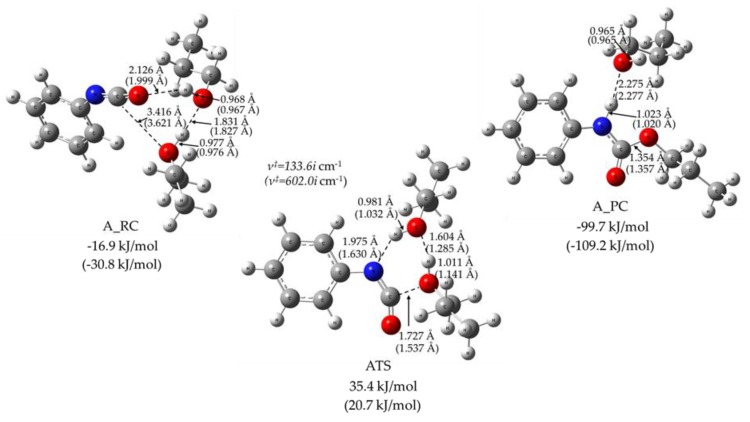
Reactive complex (RC), transition state structure (TS) and product complex (PC) structures (obtained at a B3LYP/6-31G(2df,p) level of theory from the G4MP2 calculation) for the alcohol excess reaction mechanism of urethane bond formation in solvent 1-PrOH or THF (in parenthesis). The relative zero-point corrected energies are also presented in kJ/mol.

In line with the theoretical and experimental work of Raspoet et al. [8], a reactive complex of the alcohol excess reaction (A_RC) had been characterized and its structure is shown in Figure 4. This structure is stabilized by three strong hydrogen bonds between the molecular moieties, and the energy gain of the complex formation is 16.9 kJ/mol in PrOH medium (values obtained in propanol solvent will be discussed further). In this concerted mechanism, the transition state structure (ATS in Figure 4) is a six centered structure. In ATS, the positively charged hydrogen of PrOH shifts to the electron rich nitrogen of the PhNCO, while the NCO group is being bent, activating the carbon for the formation of a new C–O bond, while the other PrOH and the hydrogen of this alcohol’s oxygen is transferred to the other alcohol in the same time. Due to the complex interaction network, the transition state energy is only 35.4 kJ/mol above the reactant level, which is consistent with the theoretical value of 27.0 kJ/mol (obtained at the MP2/6-311++G(d,p) or MP2/6-31G(d,p) level of theory) reported by Raspoet et al. [8] for methanol and hydrogen isocyanate. As a result of the IRC calculation, the product complex (A_PC) was also localized and the relevant structural parameters are displayed in Figure 4. As is seen, the urethane bond formed is strongly hydrogen bonded to the oxygen of the remaining PrOH. This exothermic reaction releases 99.7 kJ/mol energy to form A_PC. Interestingly, the relative energies of these stationary points become significantly lower by the replacement of the solvent of PrOH to THF. Obviously, the catalytic effect of the second alcohol can only be manifested when enough PrOH dimer is accessible for the urethane formation reaction.

Despite of intensive use of PhNCO as a proxy in the mechanistic studies for the urethane formation, the physicochemical properties of liquid PhNCO are scarcely mentioned in the literature. For example, only a schematic representation of the intermolecular interactions between PhNCO molecules can be found in the work of Baev [31], with an enthalpy of vaporization value (Δ_vap_*H°* = 46.5 ± 0.3 kJ/mol), while neither the viscosity or liquid structure of PhNCO were never reported to the best of our knowledge. This Δ_vap_*H* value is similar to that of 1-propanol (Δ_vap_*H°* = 47.5 kJ/mol) [32]. On the other hand, the kinematic viscosity of PhNCO is 0.96 mm^2^/s (298 K) according to our measurement, which is about 2.76 times smaller than that of 1-PrOH (2.65 mm^2^/s at 298 K). Due to the recent development of an accurate GAFF-based force field [33] for isocyanate compounds, the structural elucidation of PhNCO liquid is expected to come. Until then, as supported by the above-mentioned Δ_vap_*H°* [31] and interaction energy [19] of PhNCO being similar to those of propanol, one might hypothesize that the PhNCO dimers are stable enough to act as a reactant for the urethane formation under isocyanate excess.

**Figure 5:**
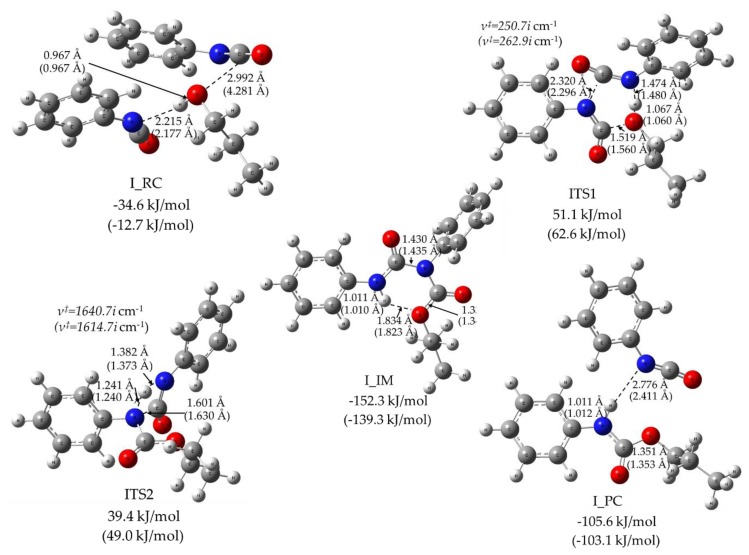
Reactive complex (RC), transition state structure (TS), intermediate (IM) and product complex (PC) structures (obtained at the B3LYP/6 31G(2df,p) level of theory from the G4MP2 calculation) for the isocyanate excess reaction mechanism of urethane bond formation in solvent 1-PrOH or THF (in parenthesis). The relative zero-point corrected energies are also presented in kJ/mol.

The reactive potential energy profile of two phenyl isocyanate molecules with PrOH is shown in Figure 3. The reactive complex (I_RC) is stabilized with a hydrogen bond between the nitrogen of one of the PhNCOs and the hydroxyl of the PrOH molecule, as shown in Figure 5. In addition, the lone pairs of the hydroxyl point towards the positively charged carbon atom of the NCO group in the second PhNCO with a distance of 2.992 Å. These interactions can significantly reduce the relative energy of the reactive complex (−34.6 kJ/mol) compared to that of the reactants. The six‑centered transition state structure (ITS1) resulted in the formation of allophanate (I_IM), which has two synchronized bond forming components that are combined with hydrogen-abstraction motion, as shown in Figure 5. In that case, both isocyanate groups are bent, and a long, new C–N bond is being formed between the isocyanate groups (2.320 Å), while the critical distance between the alcohol’s oxygen and the isocyanato carbon is extremely small (1.519 Å). In the hydrogen abstraction component of the motion along the reaction coordinate, the moving hydrogen is attacked by the nitrogen of the isocyanato group from relatively large distance (r_H‑N_ = 1.474 Å) and the O–H length is slightly elongated (r_O‑H_ = 1.067 Å). This motion also leads to the formation of a new C=O bond with a distance of 1.337 Å. ITS1 is 51.1 kJ/mol higher in energy compared to the energy level of the reactants (PhNCO dimer and PrOH) and it is 15.7 kJ/mol higher in relative energy than ATS in the case of the alcohol excess mechanism.

As IRC calculation started from ITS1 confirmed, I_RC and I_IM are connected through ITS1. The allophanate formed (I_IM, propyl *N,N’*-diphenylallophanate) is a thermodynamically stable intermediate of this potential energy surface with the corresponding relative zero-point energy of—152.3 kJ/mol. In its planar central structure, a strong intramolecular hydrogen bond can be found with a short H–O distance (r_NH-O_ = 1.834 Å). According to our B3LYP/6-31G(2df,p) calculation, N–H bond stretching mode and its rocking mode can be seen as intensive IR peaks at 3547.7 cm^−1^ and 1574.2 cm^−1^, respectively. Furthermore, an additional four IR wavenumbers with high intensities can be assigned to the allophanate functional group. Symmetric and asymmetric C=O stretch modes are at 1761.2 cm^−1^ and 1713.7 cm^−1^, respectively. The remaining two complex vibrational motions of the allophanate ire at 1367.1 cm^−1^ and 1213.1 cm^−1^. These IR spectral data may be used to monitor the components that take part in the reaction [34], although assignment of these peaks can be difficult due to the multicomponent reaction mixtures, as well as the overlap amongst the IR peaks corresponding to similar functional groups (e.g., allophanate, biuret and urethane). Proper peak assignment for allophanates is still under debate [35].

Nevertheless, the allophanate intermediate can further react through transition state ITS2, leading to the urethane–phenyl isocyanate complex I_PC. As can be seen from Figure 5, ITS2 is a tight, four‑centered transition state corresponding to a hydrogen shift from one of the allophanate nitrogens to the other. Comparing the relative energy of ITS1 and ITS2, ITS1 is found to be an energetic bottleneck of this reaction’s channel, since all thermodynamic parameters are higher for ITS1 than for ITS2 by at least 11.8 kJ/mol, as shown in Table 2. In contrast to the propanol excess mechanism, solvent change (from PrOH to THF) increased the relative energy, enthalpy and Gibbs free energy values in the isocyanate excess mechanism, as also seen from Table 2.

Allophanate formation in isocyanate excess has been already reported [36], although the previously proposed reaction mechanism starts from a covalently bonded, cyclic isocyanate dimer (uretdione) which then reacts with alcohol to give allophanate. Allophanate can then decompose to urethane and isocyanate. In contrast to that, our proposed mechanism only assumes the formation of the non-covalent dimer, which can react with alcohol through a low-lying, six-centered transition state to form an allophanate intermediate. This transition state is structurally similar to the proposed one at alcohol excess.

## 4. Conclusions

We conclude, that based on theoretical and experimental results, urethane formation can occur with the active participation of three molecules. One of these molecules, originating from either the excess alcohol or isocyanate, corresponds to self-catalysis. Our new findings indicate that, besides the alcohol-catalyzed route which had already been discussed in the literature and verified by this study, an isocyanate-catalyzed mechanism can also exist. This route, in contrast to the one-step alcohol-catalyzed mechanism, includes two consecutive reactions and the formation of an allophanate intermediate. The key step of the new mechanism is the 1,3-H shift between the nitrogens of the allophanate. The potential energy surface (PES) highly depends on the applied solvent. This agrees with the well-known solvent dependence of the reaction kinetics of urethane formation. The experimental findings, i.e., lower activation energies for either the alcohol or the isocyanate-excess reactions compared to the stoichiometric reaction, also suggest that both self-catalytical pathways could be feasible. Considering the importance of catalysis in polyurethane synthesis, molecular understanding the role of the third molecule in the reaction mechanism of urethane formation gives a new direction to the design of a better catalyst.

## Figures and Tables

**Table 1:** Experimental reaction rate constants (*k_A_*, *k_S_* and *k_I_*) at different temperatures, Arrhenius activation energies (*E_a_*) and pre-exponential factors (*A*). *E_a_* and *A* values were obtained by the method of least squares. For [NCO]_0_/[OH]_0_ = 0.005, data are taken from [11]. (n.m. = not measured).

Temperature, K	Alcohol Excess [NCO]_0_/[OH]_0_ = 0.005	Stoichiometric Ratio [NCO]_0_/[OH]_0_ = 1	Isocyanate Excess [NCO]_0_/[OH]_0_ = 20
	*k_A_* × 10^5^, M^–1^ s^–1^	*k_S_* × 10^5^, M^–1^ s^–1^	*k_I_* × 10^5^, M^–1^ s^–1^
303	*n.m.*	1.76 ± 0.18	0.52 ± 0.04
313	0.16 ± 0.01	3.72 ± 0.32	0.91 ± 0.07
323	0.23 ± 0.01	7.41 ± 0.60	1.55 ± 0.11
333	0.33 ± 0.02	*n.m.*	*n.m.*
***E_a_,* kJ mol^–1^**	30.4 ± 1.6	58.6 ± 6.0	44.2 ± 4.5
***A*, M^–1^ s^–1^**	18.8 ± 1.0	234113 ± 23971	214.9 ± 21.9

**Table 2:** G4MP2 thermochemical properties calculated in 1-propanol (PrOH) and in tetrahydrofuran (THF), including zero-point corrected relative energies (Δ*E*_0_), relative enthalpies (Δ*H*(T)) and relative Gibbs free energies (Δ*G*(T,P)) at *T* = 298.15 K, and *P* = 1 atm. (A) according to alcohol excess, and according to isocyanate excess (I). All values are in kJ/mol.

Pathway	Species	Δ*E*_0_		Δ*H*(T)		Δ*G*(T,P)	
		PrOH	THF	PrOH	THF	PrOH	THF
**Alcohol Excess (A)**	PhNCO + 2 PrOH	0	0	0	0	0	0
	A_RC	−17.0	−30.8	−14.0	−27.2	25.1	50.5
	ATS	35.4	20.7	32.7	17.0	91.4	119.2
	A_PC	−99.7	−109.3	−100.9	−109.8	−47.4	−17.2
**Isocyanate Excess (I)**	2 PhNCO + PrOH	0	0	0	0	0	0
	I_RC	−34.6	−12.7	−33.0	−11.1	−12.7	36.9
	ITS1	51.1	62.6	44.0	55.7	62.6	141.3
	I_IM	−152.3	−139.3	−160.1	−147.2	−139.3	−59.0
	ITS2	39.4	49.0	31.5	41.2	49.0	129.4
	I_PC	−105.6	−103.1	−109.2	−106.4	−103.1	−38.4

## Supplementary Materials

The following are available online at https://www.mdpi.com/2073-4360/11/10/1543/s1.

## References

[1] Boros R.Z., Farkas L., Nehéz K., Viskolcz B., Szőri M. (2019). An Ab Initio Investigation of the 4,4′-Methlylene Diphenyl Diamine (4,4′-MDA) Formation from the Reaction of Aniline with Formaldehyde. Polymers.

[2] Davis T.L., Farnum J.M. (1934). Relative Velocities of Reaction of Alcohols with Phenyl Isocyanate. J. Am. Chem. Soc..

[3] Baker J.W., Holdsworth J.B. (1947). The Mechanism of Aromatic Side-chain Reactions with Special Reference to the Polar Effects of Substituents. Part XIII. Kinetic Examination of the Reaction of Aryl Isocyanates with Methyl Alcohol. J. Chem. Soc..

[4] Baker J.W., Gaunt J. (1949). The Mechanism of the Reaction of Aryl Isocyanates with Alcohols and Amines. Part III. The “Spontaneous” Reaction of Phenyl Isocyanate with Various Alcohols. Further Evidence Relating to the Anomalous Effect of Dialkylanilines in the Base-catalysed Reaction. J. Chem. Soc..

[5] Baker J.W., Gaunt J. (1949). The Mechanism of the Reaction of Aryl Isocyanates with Alcohols and Amines. Part, V. Kinetic Investigations of the Reaction between Phenyl Isocyanate and Methyl and Ethyl Alcohols in Benzene Solution. J. Chem. Soc..

[6] Ephraim S., Woodward A.E., Mesrobian R.B. (1958). Kinetic Studies of the Reaction of Phenyl Isocyanate with Alcohols in Various Solvents. J. Am. Chem. Soc..

[7] Lammiman S.A., Satchell R.S. (1972). The Kinetics and Mechanism of the Spontaneous Alcoholysis of *p*-Chlorophenyl Isocyanate in Diethyl Ether. The Association of Alcohols in Diethyl Ether. J. Chem. Soc. Perkin Trans..

[8] Raspoet G., Nguyen M.T. (1998). The Alcoholysis Reaction of Isocyanates Giving Urethanes: Evidence for a Multimolecular Mechanism. J. Org. Chem..

[9] Makitra R.G., Midyana G.G., Pal’chikova E.Ya., Romanyuk A.V. (2012). Solvent Effect on the Kinetics of Carbamoylation of Alcohols. Russ. J. Org. Chem..

[10] Król P., Wojturska J. (2003). Kinetic Study on the Reaction of 2,4-and 2,6-Tolylene Diisocyanate with 1-Butanol in the Presence of Styrene, as a Model Reaction for the Process that Yields Interpenetrating Polyurethane–Polyester Networks. J. Appl. Polym. Sci..

[11] López C.O.C., Fejes Z., Viskolcz B. (2019). Microreactor Assisted Method for Studying Isocyanate–Alcohol Reaction Kinetics. J. Flow Chem..

[12] Çoban M., Aylin F., Konuklar S. (2011). A Computational Study on the Mechanism and the Kinetics of Urethane Formation. Comput. Theor. Chem..

[13] Kössl F., Lisaj M., Kozich V., Heyne K., Kühn O. (2015). Monitoring the Alcoholysis of Isocyanates with Infrared Spectroscopy. Chem. Phys. Lett..

[14] Wang X., Hu W., Gui D., Chi X., Wang M., Tian D., Liu J., Ma X., Pang A. (2013). DFT Study of the Proton Transfer in the Urethane Formation between 2,4-Diisocyanatotoluene and Methanol. Bull. Chem. Soc. Jpn..

[15] Somekawa K., Mitsushio M., Ueda T. (2016). Molecular Simulation of Potential Energies, Steric Changes and Substituent Effects in Urethane Formation Reactions from Isocyanates. J. Comput. Chem. Jpn..

[16] Akiyama I., Ogawa M., Takase K., Takamuku T., Yamaguchi T., Ohtori N. (2004). Liquid Structure of 1-Propanol by Molecular Dynamics Simulations and X-Ray Scattering. J. Solution Chem..

[17] Sun W., Silva W.G.D.P., van Wijngaarden J. (2019). Rotational Spectra and Structures of Phenyl Isocyanate and Phenyl Isothiocyanate. J. Phys. Chem. A.

[18] Partington J.R., Cowley E.G. (1935). Dipole Moments of Ethyl and Phenyl Isocyanates. Nature.

[19] Lenzi V., Driest P.J., Dijkstra D.J., Ramos M.M.D., Marques L.S.A. (2019). Investigation on the Intermolecular Interactions in Aliphatic Isocyanurate Liquids: Revealing the Importance of Dispersion. J. Mol. Liquids.

[20] Ramakrishnan R., Dral P.O., Rupp M., von Lilienfeld O.A. (2015). Quantum Chemistry Structures and Properties of 134 Kilo Molecules. Sci. Data.

[21] Boros R.Zs., Koós T., Cheikh W., Nehéz K., Farkas L., Viskolcz B., Szőri M. (2018). A Theoretical Study on the Phosgenation of Methylene Diphenyl Diamine (MDA). Chem. Phys. Lett..

[22] Schalk O., Townsend D., Wolf T.J.A., Holland D.M.P., Boguslavskiy A.E., Szőri M. (2018). Albert Stolow Time-Resolved Photoelectron Spectroscopy of Nitrobenzene and its Aldehydes. Chem. Phys. Lett..

[23] Curtiss L.A., Redfern P.C., Raghavachari K. (2007). Gaussian-4 Theory Using Reduced Order Perturbation Theory. J. Chem. Phys..

[24] Williams D.B.G., Lawton M. (2010). Drying of Organic Solvents: Quantitative Evaluation of the Efficiency of Several Desiccants. J. Org. Chem..

[25] Becke A.D. (1993). Density-Functional Thermochemistry. III. The Role of Exact Exchange. J. Chem. Phys..

[26] Frisch M.J., Trucks G.W., Schlegel H.B., Scuseria G.E., Robb M.A., Cheeseman J.R., Scalmani G., Barone V., Mennucci B., Petersson G.A. (2009). Gaussian 09, Revision, E.01.

[27] Dennington R.D., Keith T.A., Millam J.M. (2009). GaussView05.

[28] Gonzalez C., Schlegel H.B. (1989). An Improved Algorithm for Reaction Path Following. J. Chem. Phys..

[29] Marenich A.V., Cramer C.J., Truhlar D.G. (2009). Universal Solvation Model Based on the Generalized Born Approximation with Asymmetric Descreening. J. Chem. Theory Comput..

[30] Lide D.R. (2010). CRC Handbook of Chemistry and Physics.

[31] Baev A.K. (2013). Specific Intermolecular Interactions of Nitrogenated and Bioorganic Compounds.

[32] Majer V., Svoboda V. (1985). Enthalpies of Vaporization of Organic Compounds: A Critical Review and Data Compilation.

[33] Lenzi V., Driest P.J., Dijkstra D.J., Ramos M.M., Marques L.S. (2019). GAFF-IC: Realistic Viscosities for Isocyanate Molecules with a GAFF-based Force Field. Mol. Simulat..

[34] Al Nabulsi A., Cozzula D., Hagen T., Leitner W., Müller T.E. (2018). Isocyanurate Formation during Rigid Polyurethane Foam Assembly: A Mechanistic Study Based on in situ IR and NMR Spectroscopy. Polym. Chem..

[35] Stern T. (2017). Hierarchical fractal-structured allophanate-derived network formation in bulk polyurethane synthesis. Polym. Adv. Technol..

[36] Delebecq E., Pascault J.-P., Boutevin B., Ganachaud F. (2013). On the Versatility of Urethane/Urea Bonds: Reversibility, Blocked Isocyanate, and Non-isocyanate Polyurethane. Chem. Rev..

