# Supplementary material for: Urethane Formation with an Excess of Isocyanate or Alcohol: Experimental and Ab Initio Study
Source: Polymers (Basel). 2019 Sep 22;11(10):1543. doi: 10.3390/polym11101543 (PMC6835639; doi:10.3390/polym11101543)
Supplement: Supplementary file 1 [file polymers-11-01543-s001.pdf]

polymers

Supporting Information

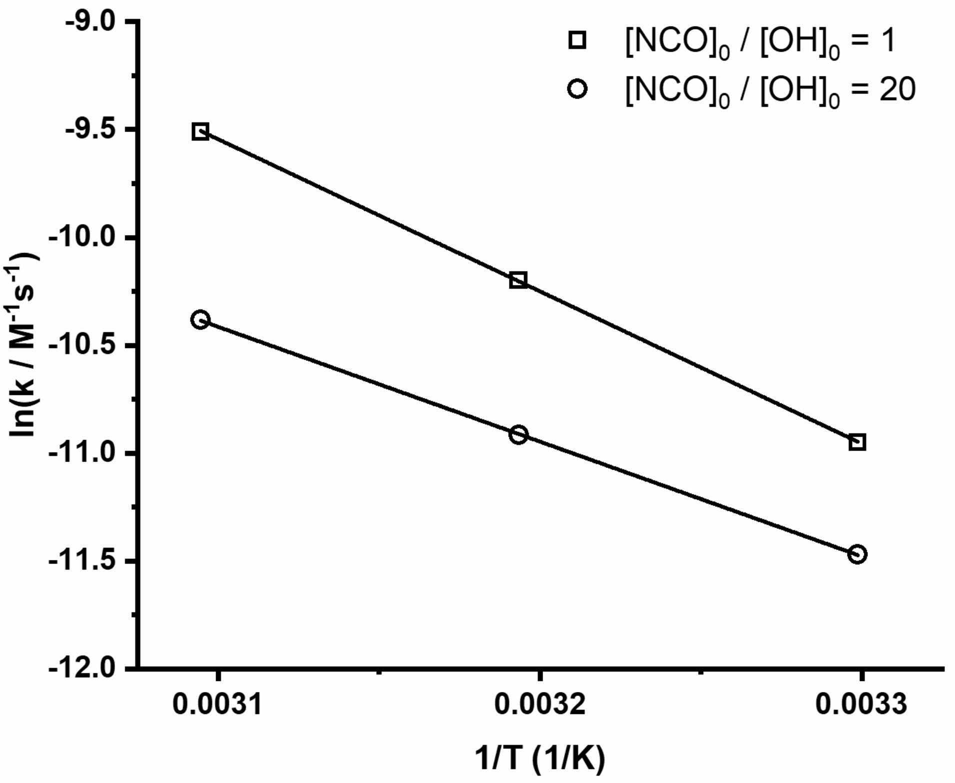

Figure S1. Arrhenius plots for the urethane formation in the reaction condition of the stochiometric and isocyanate excess over 303–323 K.

Table S1. Calculated SMD solvated B3LYP/6-31G(2df,p) geometries in Cartesian coordinates of the stationary points for the reaction system studied. The calculated G4MP2(SMD=PrOH) thermochemical properties at T=298.15 K and P=1 atm is given in Hartree.

Species	G4MP2 results (E_0, H(T), G(T,P))	Symbols	Atomic Number	Coordinates (Angstroms)		
				X	Y	Z
PrOH	-194.063935<br>-194.057346<br>-194.091806	C	6	-2.909361	-0.665201	0.165039
		H	1	-2.683684	-1.718392	0.395247
		H	1	-2.834971	-0.102905	1.109171
		O	8	-1.992133	-0.152946	-0.800301
		H	1	-1.104473	-0.232548	-0.433004
		C	6	-4.318200	-0.548058	-0.398087
		H	1	-4.363109	-1.098766	-1.346149
		H	1	-4.513986	0.505211	-0.635608
		C	6	-5.379676	-1.075698	0.567957
		H	1	-6.383150	-0.978051	0.141314
		H	1	-5.218532	-2.135295	0.798310
		H	1	-5.366744	-0.524181	1.515293
PhNCO	-399.180454<br>-399.172345<br>-399.212909	C	6	1.363993	-0.453906	1.008670
		O	8	1.904422	-1.463649	1.271945
		N	7	0.712696	0.498113	0.677208

		C	6	0.707561	1.893029	0.747436
		C	6	-0.358843	2.582332	0.166917
		C	6	1.740074	2.593490	1.381367
		C	6	-0.391063	3.972739	0.222270
		H	1	-1.149703	2.023300	-0.320265
		C	6	1.695932	3.982440	1.429775
		H	1	2.562957	2.046030	1.828564
		C	6	0.633089	4.677307	0.852143
		H	1	-1.221500	4.504693	-0.230168
		H	1	2.497557	4.523187	1.922307
		H	1	0.604502	5.760921	0.893367
PrOH dimer	-388.132251<br>-388.118752<br>-388.172969	O	8	-1.333516	-0.176214	0.628705
		H	1	-1.075373	0.325058	-0.168265
		C	6	-2.589551	0.332829	1.060820
		H	1	-2.522688	1.410621	1.282040
		H	1	-2.819483	-0.172336	2.007771
		C	6	-3.710872	0.084220	0.052601
		H	1	-3.447691	0.579050	-0.891626
		H	1	-3.759185	-0.991808	-0.157905
		C	6	-5.068367	0.588020	0.546207
		H	1	-5.046383	1.666263	0.743892
		H	1	-5.853298	0.403498	-0.194651
		H	1	-5.364684	0.087059	1.475380
		O	8	-0.585639	1.445589	-1.549899
		H	1	-0.008010	0.927510	-2.125033
		C	6	0.171865	2.558356	-1.052456
		H	1	-0.539557	3.163688	-0.480466
		H	1	0.525151	3.169551	-1.894722
		C	6	1.340934	2.131549	-0.171348
		H	1	2.016506	1.497965	-0.761697
		H	1	0.951723	1.509242	0.643117
		C	6	2.109333	3.328999	0.391183
		H	1	2.944515	3.000723	1.018074
		H	1	2.520543	3.951942	-0.411629
		H	1	1.461382	3.964425	1.006086
PhNCO dimer	-798.363714<br>-798.345862<br>-798.41869	H	8	-2.897174	2.480931	1.298884
		C	6	-3.085696	1.395122	0.890758
		N	7	-3.416003	0.319057	0.474667
		C	6	-2.881243	-0.907868	0.075327
		C	6	-3.759099	-1.905265	-0.353667
		C	6	-3.255237	-3.138367	-0.757417
		C	6	-1.883062	-3.380598	-0.735633
		C	6	-1.011492	-2.379723	-0.305890
		C	6	-1.502009	-1.143576	0.100259
		H	1	-4.824463	-1.704739	-0.366355
		H	1	-3.940670	-3.910888	-1.090165
		H	1	-1.494103	-4.342734	-1.051483
		H	1	0.058640	-2.558353	-0.285646
		H	1	-0.829567	-0.361093	0.435467
		O	8	3.057360	4.329848	-1.626033
		C	6	2.875225	3.411473	-0.916291
		N	7	2.624868	2.564325	-0.103445

		C	6	2.786341	1.196469	0.126054
		C	6	2.285082	0.659672	1.314036
		C	6	2.432917	-0.700895	1.570971
		C	6	3.076950	-1.526607	0.650314
		C	6	3.574911	-0.983254	-0.534584
		C	6	3.433961	0.373825	-0.802623
		H	1	1.789925	1.312483	2.023994
		H	1	2.045384	-1.113448	2.496594
		H	1	3.194517	-2.585200	0.855957
		H	1	4.078412	-1.618449	-1.256098
		H	1	3.819387	0.803114	-1.721254
A_RC	-787.319160<br>-787.296413<br>-787.376306	O	8	-0.934415	-0.173206	-2.180401
		C	6	0.019218	-0.678599	-1.703657
		N	7	1.022207	-1.229275	-1.362012
		C	6	1.813931	-1.610693	-0.278436
		C	6	2.971553	-2.348717	-0.532715
		C	6	3.780868	-2.742412	0.528832
		C	6	3.440725	-2.402734	1.836991
		C	6	2.282985	-1.663864	2.081025
		C	6	1.462963	-1.263761	1.031036
		H	1	3.224521	-2.605877	-1.555036
		H	1	4.679936	-3.316204	0.329507
		H	1	4.073882	-2.711000	2.662186
		H	1	2.012965	-1.395207	3.097244
		H	1	0.562038	-0.685849	1.208052
		O	8	-1.358009	0.747123	1.077827
		H	1	-1.387062	1.440447	0.389647
		C	6	-2.692497	0.465385	1.484824
		H	1	-3.164650	1.366836	1.907830
		H	1	-2.618469	-0.268158	2.297360
		C	6	-3.562138	-0.090351	0.358214
		H	1	-3.609421	0.651333	-0.449695
		H	1	-3.073746	-0.980325	-0.058225
		C	6	-4.976155	-0.436531	0.829275
		H	1	-5.491005	0.444940	1.229231
		H	1	-5.582310	-0.828705	0.006046
		H	1	-4.957908	-1.196542	1.619371
		O	8	-1.378460	2.559499	-1.059024
		H	1	-1.224271	1.885180	-1.735627
		C	6	-0.323615	3.527022	-1.137168
		H	1	-0.566024	4.288281	-0.387606
		H	1	-0.343023	4.014654	-2.122192
		C	6	1.054521	2.932109	-0.866026
		H	1	1.254938	2.146129	-1.606574
		H	1	1.043025	2.447454	0.118060
		C	6	2.160764	3.988092	-0.919846
		H	1	3.140262	3.540693	-0.723100
		H	1	2.206365	4.469060	-1.903833
		H	1	1.997756	4.773753	-0.172917
ATS	-787.299226<br>-787.278659	O	8	0.365314	-2.202092	-1.127278
		C	6	0.179391	-1.145284	-0.595824

	-787.351062	N	7	-0.634665	-0.361355	-0.008655
		C	6	-1.986291	-0.728631	0.167018
		C	6	-2.800360	0.188946	0.848174
		C	6	-4.146863	-0.077924	1.068771
		C	6	-4.713507	-1.267848	0.613289
		C	6	-3.912038	-2.184467	-0.064958
		C	6	-2.562218	-1.925794	-0.289645
		H	1	-2.358279	1.115571	1.200487
		H	1	-4.755142	0.648697	1.598707
		H	1	-5.764199	-1.477475	0.784209
		H	1	-4.338817	-3.115466	-0.426041
		H	1	-1.955382	-2.650020	-0.818310
		O	8	1.645743	-0.236563	-0.667581
		H	1	1.603733	0.524586	-0.003996
		C	6	2.885286	-0.981326	-0.581902
		H	1	3.667988	-0.312016	-0.952694
		H	1	2.776695	-1.810160	-1.284282
		C	6	3.180140	-1.469855	0.828463
		H	1	3.193421	-0.610223	1.509288
		H	1	2.366874	-2.128215	1.155958
		C	6	4.515687	-2.214001	0.893336
		H	1	5.347490	-1.567350	0.591838
		H	1	4.715657	-2.562360	1.911250
		H	1	4.516406	-3.089625	0.234347
		O	8	1.071421	1.592944	1.066818
		H	1	0.199053	1.169394	0.919067
		C	6	0.998633	2.984575	0.719796
		H	1	1.992563	3.395007	0.927752
		H	1	0.287324	3.482985	1.391944
		C	6	0.613771	3.225012	-0.736432
		H	1	-0.367201	2.771737	-0.926225
		H	1	1.331799	2.708463	-1.385221
		C	6	0.574134	4.716065	-1.077893
		H	1	0.293274	4.870783	-2.124498
		H	1	-0.154550	5.248471	-0.455745
		H	1	1.551598	5.187289	-0.922497
A_PC	-787.350669<br>-787.329532<br>-787.403904	O	8	-0.077916	-2.336124	0.810103
		C	6	-0.243619	-1.202580	0.391877
		N	7	0.713885	-0.343142	-0.049179
		C	6	2.096937	-0.569335	-0.154325
		C	6	2.863684	0.453194	-0.735345
		C	6	4.238436	0.310372	-0.874887
		C	6	4.877912	-0.850519	-0.441130
		C	6	4.116809	-1.863620	0.135965
		C	6	2.737157	-1.736024	0.285186
		H	1	2.366976	1.355443	-1.076105
		H	1	4.811209	1.113690	-1.327758
		H	1	5.951268	-0.962224	-0.551464
		H	1	4.597851	-2.773872	0.480938
		H	1	2.160749	-2.529258	0.736358
		O	8	-1.454167	-0.600386	0.303651
		H	1	-0.571509	2.253442	-1.830345

		C	6	-2.601288	-1.385060	0.699087
		H	1	-3.346380	-0.644546	1.001308
		H	1	-2.336263	-1.994346	1.566797
		C	6	-3.118285	-2.245030	-0.446947
		H	1	-3.316687	-1.599902	-1.311152
		H	1	-2.335773	-2.952183	-0.743422
		C	6	-4.387555	-3.002411	-0.050891
		H	1	-5.191930	-2.313421	0.231361
		H	1	-4.750227	-3.615344	-0.881966
		H	1	-4.204756	-3.668705	0.799830
		O	8	-0.015711	2.313234	-1.043186
		H	1	0.378555	0.567934	-0.371738
		C	6	-0.518475	3.371282	-0.217158
		H	1	-0.448522	4.326362	-0.757092
		H	1	0.164427	3.423812	0.637569
		C	6	-1.947816	3.128814	0.253729
		H	1	-1.987313	2.165537	0.775365
		H	1	-2.600504	3.041893	-0.625443
		C	6	-2.452406	4.248241	1.167026
		H	1	-3.480950	4.058020	1.489830
		H	1	-1.832268	4.337114	2.066493
		H	1	-2.438057	5.217827	0.655851
I_RC	-992.440669<br>-992.415771<br>-992.501672	O	8	-4.065707	0.984144	-1.715563
		C	6	-3.133986	1.542174	-1.266971
		N	7	-2.288288	2.203497	-0.728741
		C	6	-0.904684	2.387968	-0.708738
		C	6	-0.352323	3.145606	0.326681
		C	6	1.024375	3.348494	0.373934
		C	6	1.851438	2.804684	-0.607387
		C	6	1.293068	2.055026	-1.643178
		C	6	-0.080412	1.843118	-1.700836
		H	1	-1.005307	3.564115	1.084192
		H	1	1.448714	3.935594	1.181781
		H	1	2.923405	2.966113	-0.568419
		H	1	1.929158	1.629513	-2.412463
		H	1	-0.519972	1.258611	-2.501349
		O	8	0.259581	0.588093	2.769787
		C	6	0.619882	-0.060363	1.861913
		N	7	0.855076	-0.729073	0.887539
		C	6	1.947178	-1.359078	0.273186
		C	6	1.750324	-1.947799	-0.977223
		C	6	2.815113	-2.581642	-1.611314
		C	6	4.069080	-2.631563	-1.005638
		C	6	4.256622	-2.041492	0.244425
		C	6	3.201987	-1.404622	0.889527
		H	1	0.770150	-1.899338	-1.437373
		H	1	2.660007	-3.037874	-2.583498
		H	1	4.895994	-3.126910	-1.503135
		H	1	5.229787	-2.075759	0.723242
		H	1	3.341088	-0.944570	1.862192
		O	8	-1.704670	-1.065946	-0.932010
		H	1	-1.032670	-0.868251	-0.264611

		C	6	-2.540079	-2.104733	-0.416762
		H	1	-1.945961	-3.011407	-0.225782
		H	1	-3.248467	-2.342104	-1.218759
		C	6	-3.291223	-1.697386	0.847777
		H	1	-2.560882	-1.405255	1.614775
		H	1	-3.896536	-0.809228	0.629183
		C	6	-4.182700	-2.821622	1.380254
		H	1	-3.595485	-3.713079	1.629796
		H	1	-4.713011	-2.509185	2.285629
		H	1	-4.935133	-3.116341	0.639433
ITS1	-992.40818<br>-992.386459<br>-992.461264	O	8	1.776222	2.455064	-0.611020
		C	6	1.183822	1.397392	-0.646079
		N	7	1.433103	0.126469	-0.532859
		C	6	2.741287	-0.294615	-0.224237
		C	6	3.217055	-1.463750	-0.835721
		C	6	4.482143	-1.960088	-0.535530
		C	6	5.297589	-1.302604	0.384056
		C	6	4.830406	-0.142346	1.001122
		C	6	3.566191	0.359033	0.706015
		H	1	2.583729	-1.971324	-1.555301
		H	1	4.831028	-2.864359	-1.024903
		H	1	6.283928	-1.689373	0.618455
		H	1	5.453798	0.377626	1.722440
		H	1	3.211544	1.258068	1.196185
		O	8	0.517790	-2.302854	0.394672
		C	6	-0.180462	-1.441726	0.033994
		N	7	-1.168021	-0.743407	-0.273090
		C	6	-2.513609	-1.198284	-0.114568
		C	6	-3.520418	-0.419051	-0.686302
		C	6	-4.850761	-0.807948	-0.557071
		C	6	-5.181142	-1.969238	0.138293
		C	6	-4.169725	-2.742860	0.706139
		C	6	-2.836086	-2.364028	0.584998
		H	1	-3.257140	0.477969	-1.236048
		H	1	-5.629053	-0.198677	-1.004762
		H	1	-6.218561	-2.270062	0.237494
		H	1	-4.416343	-3.648652	1.250416
		H	1	-2.055105	-2.968320	1.033862
		O	8	-0.293267	1.519610	-0.982472
		H	1	-0.783632	0.611498	-0.708893
		C	6	-0.990124	2.755101	-0.620560
		H	1	-1.913251	2.729678	-1.203413
		H	1	-0.356244	3.561210	-0.989435
		C	6	-1.248993	2.861927	0.872633
		H	1	-1.835297	1.996845	1.202559
		H	1	-0.292065	2.832650	1.405277
		C	6	-1.993984	4.158152	1.202915
		H	1	-2.962243	4.202475	0.692030
		H	1	-2.179438	4.230789	2.278687
		H	1	-1.414278	5.037823	0.902181
I_IM	-992.485662<br>-992.464198	O	8	2.238080	1.758652	-0.282565
		C	6	1.147127	1.229651	-0.280325

	-992.539141	N	7	0.989360	-0.146063	-0.114643
		C	6	2.235319	-0.880880	0.003073
		C	6	2.891077	-1.308889	-1.146861
		C	6	4.090459	-2.007539	-1.031479
		C	6	4.625671	-2.275141	0.227700
		C	6	3.960572	-1.842479	1.373875
		C	6	2.761469	-1.142113	1.264146
		H	1	2.463042	-1.094351	-2.120189
		H	1	4.605105	-2.342545	-1.925958
		H	1	5.559888	-2.820000	0.315626
		H	1	4.374252	-2.048379	2.355546
		H	1	2.234656	-0.798627	2.147891
		O	8	-0.036175	-2.173830	0.005599
		C	6	-0.184512	-0.962706	-0.062116
		N	7	-1.381618	-0.327717	-0.074724
		C	6	-2.668528	-0.901573	-0.036650
		C	6	-3.747135	-0.006362	-0.099994
		C	6	-5.054751	-0.472790	-0.065884
		C	6	-5.313804	-1.839491	0.031540
		C	6	-4.243290	-2.726971	0.094870
		C	6	-2.925179	-2.274966	0.062396
		H	1	-3.551997	1.059464	-0.175998
		H	1	-5.873504	0.237922	-0.116106
		H	1	-6.334708	-2.205056	0.057972
		H	1	-4.427052	-3.794169	0.171751
		H	1	-2.105783	-2.974505	0.113587
		O	8	-0.005576	1.887736	-0.447283
		H	1	-1.353655	0.681629	-0.140521
		C	6	0.067187	3.333839	-0.604147
		H	1	-0.837095	3.580301	-1.164673
		H	1	0.943448	3.569275	-1.210932
		C	6	0.100578	4.039085	0.742472
		H	1	-0.766875	3.720557	1.332193
		H	1	0.998067	3.728741	1.288278
		C	6	0.091552	5.559697	0.568655
		H	1	-0.806816	5.895720	0.038548
		H	1	0.111456	6.059129	1.542076
		H	1	0.963909	5.900923	0.000139
ITS2	-992.412657<br>-992.391214<br>-992.465658	O	8	3.070396	-0.185858	-0.061767
		C	6	1.905931	-0.447960	0.093516
		N	7	0.848508	0.510860	-0.109783
		C	6	1.263503	1.898232	-0.193443
		C	6	1.798204	2.530467	0.927467
		C	6	2.149922	3.874315	0.842295
		C	6	1.967003	4.573850	-0.350364
		C	6	1.430158	3.929508	-1.462810
		C	6	1.074436	2.584501	-1.388039
		H	1	1.932074	1.977823	1.850226
		H	1	2.567878	4.374301	1.709494
		H	1	2.243809	5.621076	-0.411892
		H	1	1.287471	4.470154	-2.392297
		H	1	0.657972	2.070218	-2.247471

		O	8	-0.556358	0.449776	1.926254
		C	6	-0.491581	0.275933	0.734240
		N	7	-1.243784	-0.061514	-0.299764
		C	6	-2.605134	-0.390298	-0.262663
		C	6	-3.216147	-0.724929	-1.479750
		C	6	-4.564452	-1.060185	-1.523992
		C	6	-5.327801	-1.068514	-0.356758
		C	6	-4.723819	-0.737172	0.855133
		C	6	-3.374419	-0.399444	0.912040
		H	1	-2.617315	-0.716613	-2.384705
		H	1	-5.020141	-1.315783	-2.475566
		H	1	-6.380117	-1.330071	-0.391355
		H	1	-5.307161	-0.739877	1.770878
		H	1	-2.914550	-0.143180	1.857868
		O	8	1.378212	-1.611396	0.429887
		H	1	-0.025564	0.154559	-0.915533
		C	6	2.294946	-2.731542	0.616013
		H	1	1.768879	-3.384629	1.314847
		H	1	3.205786	-2.355602	1.087045
		C	6	2.579205	-3.431399	-0.703284
		H	1	1.627093	-3.725722	-1.159358
		H	1	3.064695	-2.726148	-1.386878
		C	6	3.469474	-4.658858	-0.495707
		H	1	2.995393	-5.387002	0.172039
		H	1	3.665639	-5.157949	-1.449487
		H	1	4.435089	-4.381557	-0.058324
I_PC	-992.467862<br>-992.444786<br>-992.525723	O	8	-3.157751	1.281544	0.041121
		C	6	-2.205433	0.634874	-0.356711
		N	7	-0.939237	1.096361	-0.563406
		C	6	-0.439721	2.398948	-0.393218
		C	6	-1.171418	3.444674	0.183463
		C	6	-0.583798	4.700938	0.318453
		C	6	0.720693	4.937991	-0.107231
		C	6	1.446127	3.893797	-0.679549
		C	6	0.873946	2.635790	-0.824100
		H	1	-2.182702	3.273490	0.520425
		H	1	-1.161918	5.502584	0.767650
		H	1	1.166296	5.920567	0.004710
		H	1	2.464131	4.055812	-1.019358
		H	1	1.439665	1.826070	-1.275114
		O	8	-0.653844	-3.421242	-0.525894
		C	6	0.199927	-2.678962	-0.210559
		N	7	0.981309	-1.829500	0.124642
		C	6	2.346751	-1.670522	0.385008
		C	6	2.788083	-0.445869	0.889711
		C	6	4.141835	-0.259117	1.155527
		C	6	5.054905	-1.285310	0.921217
		C	6	4.606951	-2.506420	0.416974
		C	6	3.257536	-2.705843	0.146828
		H	1	2.069721	0.345249	1.070867
		H	1	4.480609	0.693957	1.548121
		H	1	6.108898	-1.136074	1.129806

H	1	5.310947	-3.311201	0.231847
H	1	2.902430	-3.652291	-0.246801
O	8	-2.252090	-0.677201	-0.675692
H	1	-0.285417	0.410789	-0.917277
C	6	-3.522345	-1.347393	-0.504320
H	1	-3.488521	-2.175471	-1.216616
H	1	-4.325129	-0.664131	-0.792442
C	6	-3.707840	-1.854103	0.919506
H	1	-2.866416	-2.508897	1.174012
H	1	-3.678860	-1.002462	1.608224
C	6	-5.029307	-2.609973	1.074847
H	1	-5.075362	-3.475640	0.403990
H	1	-5.149676	-2.976037	2.099226
H	1	-5.886305	-1.965647	0.847890

Table S2. Calculated SMD solvated B3LYP/6-31G(2df,p) geometries in Cartesian coordinates of the stationary points for the reaction system studied. The calculated G4MP2(SMD=THF) thermochemical properties at T=298.15K and P=1atm is given in Hartree.

Species	G4MP2 Results (E_0, H(T), G(T,P))	Symbols	Atomic Number	Coordinates (Angstroms)		
				X	Y	Z
PrOH	-194.060899<br>-194.054295<br>-194.088799	C	6	-2.907577	-0.664746	0.163849
		H	1	-2.685087	-1.719296	0.395320
		H	1	-2.837448	-0.104684	1.110715
		O	8	-1.993977	-0.151616	-0.800223
		H	1	-1.106685	-0.232240	-0.433808
		C	6	-4.317196	-0.548280	-0.398248
		H	1	-4.361646	-1.098591	-1.346080
		H	1	-4.512047	0.504598	-0.636496
		C	6	-5.379000	-1.075387	0.568026
		H	1	-6.382496	-0.977963	0.141685
		H	1	-5.218461	-2.134866	0.799182
		H	1	-5.366397	-0.523759	1.515260
PhNCO	-399.184895<br>-399.176788<br>-399.217348	C	6	1.361927	-0.457303	1.007885
		O	8	1.897779	-1.467634	1.270991
		N	7	0.713993	0.498649	0.675929
		C	6	0.709986	1.892343	0.747574
		C	6	-0.357000	2.582052	0.167365
		C	6	1.741405	2.595410	1.381216
		C	6	-0.390854	3.972161	0.222739
		H	1	-1.146794	2.021374	-0.319403
		C	6	1.695808	3.984255	1.429670
		H	1	2.565409	2.050170	1.828859
		C	6	0.632414	4.678123	0.852387
		H	1	-1.221912	4.503149	-0.229472
		H	1	2.496876	4.525717	1.922112
		H	1	0.602637	5.761562	0.893680
PrOH dimer	-388.127484<br>-388.113924<br>-388.168710	O	8	-1.356571	-0.110161	0.607761
		H	1	-1.114418	0.375698	-0.199955
		C	6	-2.613096	0.381218	1.040000
		H	1	-2.577298	1.468990	1.223236
		H	1	-2.815730	-0.093239	2.009300
		C	6	-3.749818	0.067412	0.065775

		H	1	-3.519934	0.531595	-0.902474
		H	1	-3.774246	-1.016349	-0.103546
		C	6	-5.110045	0.556207	0.567751
		H	1	-5.111226	1.641256	0.725407
		H	1	-5.905319	0.325523	-0.148633
		H	1	-5.375574	0.083944	1.520927
		O	8	-0.545336	1.487540	-1.606629
		H	1	0.039409	0.961529	-2.166739
		C	6	0.223706	2.577628	-1.078143
		H	1	-0.488962	3.188729	-0.514184
		H	1	0.607125	3.195882	-1.902417
		C	6	1.363118	2.116785	-0.175008
		H	1	2.043846	1.480339	-0.756952
		H	1	0.940525	1.488776	0.617659
		H	6	2.138519	3.291190	0.425776
		H	1	2.948279	2.937694	1.071635
		H	1	2.585175	3.917087	-0.355334
		H	1	1.485539	3.930506	1.030966
PhNCO dimer	-798.37246<br>-798.354615<br>-798.428003	O	8	-2.905651	2.486196	1.314125
		C	6	-3.093611	1.407377	0.892285
		N	7	-3.423301	0.335584	0.460435
		C	6	-2.888163	-0.886911	0.051684
		C	6	-3.765952	-1.877390	-0.394754
		C	6	-3.264033	-3.107634	-0.808654
		C	6	-1.893026	-3.355600	-0.780204
		C	6	-1.021013	-2.362785	-0.333600
		C	6	-1.509888	-1.129413	0.082707
		H	1	-4.830189	-1.671879	-0.412158
		H	1	-3.950027	-3.873687	-1.154672
		H	1	-1.505593	-4.315578	-1.103976
		H	1	0.048220	-2.545178	-0.307959
		H	1	-0.836000	-0.354011	0.430633
		O	8	2.853919	4.265650	-1.696417
		C	6	2.745635	3.363124	-0.954703
		N	7	2.575442	2.530237	-0.105043
		C	6	2.780325	1.173004	0.144341
		C	6	2.371957	0.657408	1.377123
		C	6	2.563260	-0.692747	1.657352
		C	6	3.159564	-1.530967	0.716266
		C	6	3.565243	-1.010159	-0.512962
		C	6	3.379599	0.336568	-0.804824
		H	1	1.913664	1.320713	2.101804
		H	1	2.247060	-1.087701	2.617072
		H	1	3.311382	-2.581413	0.939774
		H	1	4.031458	-1.654957	-1.250680
		H	1	3.693780	0.747407	-1.758295
A_RC	-787.318417<br>-787.295744<br>-787.375725	O	8	-0.047333	0.271049	-2.409349
		C	6	0.577569	-0.535794	-1.814721
		N	7	1.250825	-1.402293	-1.351095
		C	6	1.741341	-2.039724	-0.213048
		C	6	2.604098	-3.124682	-0.382066
		C	6	3.111586	-3.776699	0.737551

		C	6	2.762486	-3.350697	2.017758
		C	6	1.899712	-2.266028	2.174972
		C	6	1.381889	-1.602885	1.066927
		H	1	2.867007	-3.443308	-1.384275
		H	1	3.782221	-4.619285	0.606008
		H	1	3.160386	-3.860623	2.888586
		H	1	1.624250	-1.930659	3.169665
		H	1	0.706986	-0.758823	1.175588
		O	8	-0.882296	0.871162	1.184906
		H	1	-0.965565	1.536296	0.476084
		C	6	-2.191352	0.464541	1.552628
		H	1	-2.787643	1.329561	1.887434
		H	1	-2.078832	-0.198128	2.420239
		C	6	-2.936446	-0.267159	0.435520
		H	1	-3.014445	0.402314	-0.430520
		H	1	-2.334494	-1.128012	0.117797
		C	6	-4.330872	-0.729079	0.864308
		H	1	-4.955824	0.118783	1.168534
		H	1	-4.847104	-1.244171	0.047645
		H	1	-4.278273	-1.422009	1.712365
		O	8	-1.205336	2.563650	-1.015196
		H	1	-0.834881	1.951573	-1.666304
		C	6	-0.620816	3.854320	-1.214602
		H	1	-1.103035	4.514354	-0.485398
		H	1	-0.879716	4.228002	-2.216090
		C	6	0.892894	3.865429	-1.022647
		H	1	1.347738	3.166512	-1.736933
		H	1	1.125414	3.489528	-0.018827
		C	6	1.488878	5.261996	-1.214309
		H	1	2.574170	5.249052	-1.073529
		H	1	1.288379	5.646392	-2.220915
		H	1	1.068680	5.975525	-0.496221
ATS	-787.298818<br>-787.278897<br>-787.349561	O	8	0.371334	-2.036814	-1.034847
		C	6	0.297280	-0.975923	-0.461983
		N	7	-0.591468	-0.241268	0.156517
		C	6	-1.935632	-0.631645	0.246848
		C	6	-2.783932	0.208140	0.990848
		C	6	-4.135472	-0.078925	1.136360
		C	6	-4.683980	-1.214749	0.541098
		C	6	-3.853764	-2.054172	-0.198779
		C	6	-2.497310	-1.776316	-0.349086
		H	1	-2.361654	1.094190	1.455182
		H	1	-4.763086	0.589570	1.718024
		H	1	-5.739158	-1.440848	0.652644
		H	1	-4.263909	-2.943108	-0.669290
		H	1	-1.866740	-2.438866	-0.925228
		O	8	1.615036	-0.186135	-0.433852
		H	1	1.531222	0.701824	0.277743
		C	6	2.855555	-0.938199	-0.483124
		H	1	3.605424	-0.233144	-0.852322
		H	1	2.704903	-1.718986	-1.229595
		C	6	3.235477	-1.515701	0.872206

		H	1	3.310146	-0.703863	1.605364
		H	1	2.436616	-2.185060	1.211071
		C	6	4.560319	-2.278910	0.796354
		H	1	5.380906	-1.624753	0.480972
		H	1	4.822592	-2.696002	1.773106
		H	1	4.499911	-3.108464	0.083320
		O	8	0.992228	1.544560	1.084651
		H	1	0.118878	1.031751	0.885837
		C	6	0.912344	2.927449	0.677448
		H	1	1.883099	3.371795	0.919242
		H	1	0.157529	3.412981	1.306703
		C	6	0.583088	3.111857	-0.800293
		H	1	-0.369992	2.617677	-1.021839
		H	1	1.348059	2.612788	-1.407208
		C	6	0.505149	4.593448	-1.175870
		H	1	0.268135	4.713608	-2.237304
		H	1	-0.271340	5.109890	-0.600423
		H	1	1.456117	5.104420	-0.986809
A_PC	-787.348309<br>-787.327214<br>-787.401497	O	8	-0.064994	-2.360283	0.781102
		C	6	-0.229145	-1.221280	0.391323
		N	7	0.728595	-0.356768	-0.054135
		C	6	2.109034	-0.587439	-0.168936
		C	6	2.891894	0.463291	-0.674183
		C	6	4.265334	0.311716	-0.818085
		C	6	4.888185	-0.884409	-0.463617
		C	6	4.110960	-1.925232	0.037924
		C	6	2.732512	-1.790985	0.189366
		H	1	2.408588	1.394591	-0.949824
		H	1	4.850894	1.137233	-1.210717
		H	1	5.960581	-1.001892	-0.576517
		H	1	4.578812	-2.863693	0.319710
		H	1	2.137948	-2.603148	0.579223
		O	8	-1.435044	-0.600611	0.340791
		H	1	-0.331555	2.436314	-1.802095
		C	6	-2.576442	-1.384863	0.741758
		H	1	-3.298274	-0.653648	1.116314
		H	1	-2.285470	-2.047561	1.560365
		C	6	-3.156347	-2.176121	-0.424134
		H	1	-3.385561	-1.484885	-1.243825
		H	1	-2.395600	-2.873252	-0.792031
		C	6	-4.415170	-2.943114	-0.012794
		H	1	-5.199163	-2.264113	0.341899
		H	1	-4.821574	-3.507993	-0.857416
		H	1	-4.201897	-3.655193	0.792400
		O	8	0.122638	2.390497	-0.951555
		H	1	0.398374	0.563280	-0.347044
		C	6	-0.466384	3.366054	-0.081346
		H	1	-0.268706	4.376129	-0.468356
		H	1	0.067494	3.266827	0.869452
		C	6	-1.962269	3.151630	0.118225
		H	1	-2.120886	2.131544	0.485639
		H	1	-2.463956	3.220127	-0.857086

		C	6	-2.570264	4.171228	1.083939
		H	1	-3.644925	4.003808	1.205972
		H	1	-2.108372	4.103996	2.075522
		H	1	-2.432370	5.196393	0.721560
I_RC	-992.438199<br>-992.413124<br>-992.502762	O	8	-4.437108	1.783838	0.012350
		C	6	-3.501754	2.435057	-0.272212
		N	7	-2.632639	3.214227	-0.551975
		C	6	-1.278706	3.282101	-0.886234
		C	6	-0.721590	4.539636	-1.129039
		C	6	0.625563	4.642972	-1.462513
		C	6	1.417668	3.500065	-1.554676
		C	6	0.853430	2.247799	-1.311381
		C	6	-0.491826	2.127885	-0.977625
		H	1	-1.349292	5.420274	-1.053425
		H	1	1.054950	5.621623	-1.649945
		H	1	2.467631	3.583732	-1.813990
		H	1	1.464003	1.353064	-1.379042
		H	1	-0.930822	1.153344	-0.786082
		O	8	0.391677	-2.082698	3.786978
		C	6	0.800447	-2.079311	2.690520
		N	7	1.088839	-2.041278	1.517737
		C	6	2.231914	-2.245636	0.726194
		C	6	2.103385	-2.130895	-0.659476
		C	6	3.218595	-2.327281	-1.469403
		C	6	4.455209	-2.636632	-0.906943
		C	6	4.574600	-2.749597	0.478062
		C	6	3.469542	-2.555554	1.299666
		H	1	1.135261	-1.890237	-1.083962
		H	1	3.115487	-2.238182	-2.545751
		H	1	5.321030	-2.789128	-1.542107
		H	1	5.533545	-2.989896	0.925198
		H	1	3.558612	-2.641181	2.377565
		O	8	-1.174078	-1.155792	-0.396124
		H	1	-0.613106	-1.304880	0.377353
		C	6	-2.242474	-2.100698	-0.349310
		H	1	-1.845475	-3.126950	-0.315215
		H	1	-2.778731	-1.992745	-1.299094
		C	6	-3.194944	-1.867513	0.820963
		H	1	-2.630838	-1.946090	1.760768
		H	1	-3.574896	-0.840730	0.764779
		C	6	-4.357982	-2.862070	0.831375
		H	1	-3.999834	-3.895371	0.907197
		H	1	-5.025850	-2.678949	1.678997
		H	1	-4.954900	-2.784175	-0.084663
ITS1	-992.409529<br>-992.387698<br>-992.462996	O	8	1.800883	2.479486	-0.576778
		C	6	1.223837	1.424916	-0.628839
		N	7	1.441929	0.147453	-0.523290
		C	6	2.747501	-0.295599	-0.223799
		C	6	3.196503	-1.474482	-0.835284
		C	6	4.458271	-1.987506	-0.550870
		C	6	5.296936	-1.336370	0.351972
		C	6	4.856674	-0.165135	0.967522

		C	6	3.595584	0.352533	0.688349
		H	1	2.545209	-1.975776	-1.542547
		H	1	4.786685	-2.899406	-1.040019
		H	1	6.281118	-1.735775	0.573511
		H	1	5.499282	0.351539	1.674046
		H	1	3.263161	1.263763	1.170609
		O	8	0.507714	-2.252191	0.435686
		C	6	-0.169337	-1.386407	0.047693
		N	7	-1.162194	-0.699709	-0.282409
		C	6	-2.501593	-1.170224	-0.120992
		C	6	-3.510470	-0.461528	-0.776607
		C	6	-4.836211	-0.864961	-0.646951
		C	6	-5.162951	-1.971819	0.133438
		C	6	-4.151823	-2.675126	0.786126
		C	6	-2.822887	-2.280390	0.664873
		H	1	-3.252566	0.391496	-1.394660
		H	1	-5.613999	-0.310237	-1.161202
		H	1	-6.196665	-2.284471	0.232850
		H	1	-4.394807	-3.537656	1.397865
		H	1	-2.044861	-2.829440	1.183422
		O	8	-0.285052	1.558813	-1.005286
		H	1	-0.768526	0.653872	-0.735395
		C	6	-0.974424	2.781784	-0.608858
		H	1	-1.868839	2.822283	-1.235914
		H	1	-0.303816	3.592476	-0.894325
		C	6	-1.308820	2.816873	0.874163
		H	1	-1.934259	1.954382	1.130986
		H	1	-0.382189	2.732202	1.452487
		C	6	-2.035921	4.114574	1.237828
		H	1	-2.977163	4.212447	0.685667
		H	1	-2.271119	4.137164	2.305835
		H	1	-1.420406	4.991964	1.011257
I_IM	-992.486409<br>-992.464982<br>-992.539291	O	8	2.228103	1.763689	-0.307734
		C	6	1.144218	1.231560	-0.282325
		N	7	0.984737	-0.146208	-0.118320
		C	6	2.232180	-0.879221	-0.004330
		C	6	2.864848	-1.341202	-1.153498
		C	6	4.065600	-2.038025	-1.043497
		C	6	4.627160	-2.269646	0.211099
		C	6	3.986429	-1.802078	1.357326
		C	6	2.785792	-1.104369	1.251501
		H	1	2.417183	-1.154620	-2.123678
		H	1	4.560904	-2.399731	-1.938444
		H	1	5.562496	-2.813034	0.295411
		H	1	4.420296	-1.979337	2.335832
		H	1	2.277986	-0.734753	2.135747
		O	8	-0.040334	-2.170937	0.079321
		C	6	-0.188489	-0.969837	-0.046022
		N	7	-1.388769	-0.334636	-0.117733
		C	6	-2.674087	-0.906359	-0.064616
		C	6	-3.755220	-0.020244	-0.188905
		C	6	-5.061734	-0.488936	-0.144458

		C	6	-5.317453	-1.849147	0.024298
		C	6	-4.244653	-2.727724	0.147419
		C	6	-2.927837	-2.273631	0.105226
		H	1	-3.562509	1.040642	-0.321679
		H	1	-5.882110	0.214755	-0.242727
		H	1	-6.337318	-2.216598	0.058926
		H	1	-4.426251	-3.789789	0.279837
		H	1	-2.102829	-2.961800	0.202102
		O	8	-0.019758	1.894119	-0.418039
		H	1	-1.357019	0.669501	-0.228508
		C	6	0.059446	3.338665	-0.570451
		H	1	-0.846828	3.596660	-1.123473
		H	1	0.933238	3.572753	-1.180954
		C	6	0.108737	4.041883	0.777371
		H	1	-0.756461	3.730750	1.374375
		H	1	1.007849	3.721726	1.314153
		C	6	0.113039	5.563106	0.607291
		H	1	-0.785099	5.909435	0.083528
		H	1	0.143742	6.060503	1.581252
		H	1	0.984997	5.897293	0.034263
ITS2	-992.414687<br>-992.393219<br>-992.467503	O	8	3.072611	-0.195232	0.025770
		C	6	1.904062	-0.456218	0.125630
		N	7	0.850953	0.504286	-0.084716
		C	6	1.267191	1.887619	-0.182899
		C	6	1.800858	2.536749	0.929002
		C	6	2.149996	3.879957	0.827775
		C	6	1.965584	4.565568	-0.372608
		C	6	1.428860	3.906872	-1.476408
		C	6	1.076177	2.562272	-1.384539
		H	1	1.934211	1.997841	1.859524
		H	1	2.566749	4.391000	1.689026
		H	1	2.240172	5.612481	-0.446521
		H	1	1.283627	4.436068	-2.412019
		H	1	0.659290	2.037769	-2.237628
		O	8	-0.578035	0.466029	1.960723
		C	6	-0.515221	0.282838	0.777203
		N	7	-1.253971	-0.053519	-0.268837
		C	6	-2.615153	-0.380177	-0.245236
		C	6	-3.214902	-0.718841	-1.467020
		C	6	-4.562666	-1.053964	-1.523064
		C	6	-5.337459	-1.058384	-0.363463
		C	6	-4.745027	-0.722706	0.852870
		C	6	-3.396367	-0.385061	0.921734
		H	1	-2.606678	-0.713933	-2.365669
		H	1	-5.009118	-1.312932	-2.478043
		H	1	-6.389277	-1.320081	-0.407374
		H	1	-5.337182	-0.722110	1.762882
		H	1	-2.943205	-0.124990	1.869790
		O	8	1.361674	-1.633943	0.404384
		H	1	-0.037102	0.146706	-0.873828
		C	6	2.274401	-2.751900	0.594845
		H	1	1.731630	-3.426278	1.260581

		H	1	3.169466	-2.386306	1.102915
		C	6	2.610627	-3.423458	-0.727982
		H	1	1.678563	-3.717359	-1.223881
		H	1	3.112883	-2.700051	-1.379470
		C	6	3.505667	-4.646774	-0.515374
		H	1	3.016880	-5.393093	0.120870
		H	1	3.739696	-5.124820	-1.471186
		H	1	4.452864	-4.369557	-0.039510
		O	8	-3.077644	1.366905	0.083249
		C	6	-2.116017	0.705384	-0.248361
		N	7	-0.844421	1.160522	-0.467437
		C	6	-0.370551	2.481537	-0.421393
		C	6	-1.150060	3.573409	-0.017274
		C	6	-0.589370	4.848622	0.000338
		C	6	0.734753	5.061623	-0.374737
		C	6	1.507490	3.972598	-0.775575
		C	6	0.962944	2.694715	-0.801291
		H	1	-2.177242	3.416212	0.275345
		H	1	-1.204802	5.685773	0.315034
		H	1	1.158772	6.059710	-0.355851
		H	1	2.541414	4.114895	-1.073409
		H	1	1.566581	1.849884	-1.118796
		O	8	-0.305197	-3.009312	-1.942649
		C	6	0.396501	-2.373962	-1.253826
		N	7	1.027289	-1.631913	-0.541165
		C	6	2.282614	-1.600970	0.081567
		C	6	2.530349	-0.595845	1.018857
I_PC	-992.472609<br>-992.449448<br>-992.531445	C	6	3.768652	-0.533676	1.651604
		C	6	4.759682	-1.467070	1.355391
		C	6	4.506087	-2.468999	0.418697
		C	6	3.273473	-2.541603	-0.220882
		H	1	1.750513	0.121327	1.247639
		H	1	3.955213	0.248575	2.379765
		H	1	5.723368	-1.415548	1.850297
		H	1	5.271780	-3.200391	0.181949
		H	1	3.072585	-3.317202	-0.952203
		O	8	-2.139818	-0.629513	-0.473298
		H	1	-0.174357	0.449998	-0.734098
		C	6	-3.396697	-1.302239	-0.250002
		H	1	-3.374826	-2.157597	-0.930227
		H	1	-4.213097	-0.636509	-0.540178
		C	6	-3.542984	-1.755329	1.196863
		H	1	-2.679838	-2.376633	1.463504
		H	1	-3.521530	-0.874543	1.848194
		C	6	-4.841616	-2.535611	1.412595
		H	1	-4.879406	-3.430538	0.780833
		H	1	-4.933474	-2.859422	2.453876
		H	1	-5.718730	-1.922933	1.175572
